# Factor structure, measurement invariance and psychometric properties of the Quality of Life Scale WHOQOL-BREF in the Ecuadorian context

**DOI:** 10.1186/s41155-021-00194-9

**Published:** 2021-10-07

**Authors:** Sandra Lima-Castro, Paúl Arias-Medina, Alexandra Bueno-Pacheco, Eva Peña-Contreras, Mónica Aguilar-Sizer, Marcela Cabrera-Vélez

**Affiliations:** 1 Faculty of Psychology, University of Cuenca, Av. 12 de abril y Agustín Cueva, Cueca, Ecuador; 2 Faculty of Philosophy, Letters and Educational Sciences, Universidad del Azuay, Av. 24 de mayo 7-77 and Hernán Malo. Section: 01.01.981, Cuenca, Ecuador

**Keywords:** Quality of life, WHOQOL-BREF, Factor analysis, Measurement invariance, Psychometric properties, Ecuador

## Abstract

**Background:**

The short version of the World Health Organization Quality of Life questionnaire (WHOQOL-BREF) is a popular instrument used to assess quality of life. The objective of this study was to evaluate the following psychometric properties: structural validity, convergent validity, internal consistency, and measurement invariance across sex of the WHOQOL-BREF in a sample of Ecuadorian adults.

**Methods:**

We used a sample of undergraduates (*n* = 987) to assess the WHOQOL-BREF original four-factor structure, a model with correlated factors, a hierarchical model, and two models resulting from the exploratory factor analysis and exploratory graph analysis. All the models were evaluated using confirmatory factor analysis.

**Results:**

The results of the exploratory factor analysis and exploratory graph analysis suggest that the items are organized into four factors, although differently from the original version and the orthogonality assumption is not maintained. The confirmatory factor analysis shows that the original WHOQOL-BREF structure with correlated factors presents adequate psychometric properties. However, we propose a four-factor structure that has the best psychometric properties and adequate internal consistency. The results of the measurement invariance show that strict and strong invariance is achieved between men and women. Convergent validity analysis reveals moderate correlations with self-esteem, resilience, and social support.

**Conclusions:**

Despite the original version of the WHOQOL-BREF with correlated factors has acceptable psychometric properties in the Ecuadorian context, we propose a version with a different organization of its items, which is consistent with the findings of other investigations.

## Introduction

The Quality of Life (QOL) evaluation is a research line that has been promoted with emphasis from different fields: economics, medical, and social sciences (Cummins & Lau, 2006). In recent decades, it has become an essential element in the health field since it has been used to improve results and increase standards to treat and intervene in chronic diseases, disabilities, and medical conditions (Barneveld et al., 2014; Burgess & Gutstein, 2007; Oliveira et al., 2016; Sosnowski et al., 2017); yet, its study constitutes a challenge for research due to the dispersion and diversity of its conceptions, the dimensions that comprise it, challenges in its measurement, and the factors that influence it (Taillefer et al., 2003). Based on cross-cultural studies and with the objective of unifying criteria, the World Health Organization (WHO) proposed the following definition of quality of life: “the perception that an individual has of his situation in life within the cultural context and value system in which he lives and in relation to his objectives, expectations, norms and interests” (The WHOQOL Group, 1996). This definition denotes the subjective, cultural, and multidimensional nature of the QOL. From this definition, the World Health Organization Quality of Life questionnaire (WHOQOL-BREF) was developed. The instrument of generic evaluation of the quality of life stands out for its rigor in the process of translation and intercultural adaptation, allowing to obtain reliable conclusions about its equivalence in 15 cultures (Bowden & Fox-Rushby, 2003; Crocker et al., 2015). In addition, the WHOQOL-BREF questionnaire stands out among the most popular generic measurement instruments of quality of life used in clinical and epidemiological research because it allows collecting information on four domains through 26 items: physical and psychological (commonly evaluated in the clinical field), social relations, and environment. It transcends its application to other areas beyond public health (Skevington & Epton, 2018). In cross-cultural studies, it shows excellent internal consistency, good test–retest reliability, discriminant validity, and construct validity in the general population and in different groups of patients with different diseases that cause disability (Harper et al., 1998; Jang et al., 2004; Min et al., 2002; Skevington et al., 2004). Factor invariance through gender has been reported as well (Perera et al., 2018).

The international use of WHOQOL-BREF continues to expand, but the structure evaluation is required because there are conflicting results. For instance, there is research that supports the original WHO four-domain model such as a recent study in Singapore involving 3400 adults with a wide range of age and health conditions, reported an acceptable fit of the data with the original four domain model (Suárez et al., 2018), whereas in Taiwan, a sample of 1068 healthy and unhealthy adults was used, and the exploratory and confirmatory factor analysis suggested a four-domain model (Yao et al., 2002). However, other investigations could not replicate the original structure of the WHOQOL-BREF (Benitez-Borrego et al., 2014; Benítez-Borrego et al., 2016; Moreno et al., 2006; Ohaeri et al., 2007; Oliveira et al., 2016). In short, the importance of clarifying the structural factor of the questionnaire in different contexts is evident (Ohaeri et al., 2007; Perera et al., 2018; Snell et al., 2016), given the linguistic and cultural differences that can be detected even when the language is the same (Benítez-Borrego et al., 2016; Hambleton et al., 2004).

Besides, in the study of quality of life, it is essential to recognize that there are many different factors correlated with quality of life in general population and clinical samples. For example, previous researches recommended to put attention to self-esteem, as an important factor influencing the promotion of quality of life of patients with chronic disease, depression and the elderly (Hemati & Kiani, 2016; Kuehner & Buerger, 2005; Tavares et al., 2016). Other protective factors like resilience were found to be an important predictor of high levels of quality of life in cancer patients (Ristevska-Dimitrоvska et al., 2015) and people suffering from chronic pain (Yazdi-Ravandi et al., 2013). The literature also reveals that social support is beneficious to QOL in depressed patients (Kuehner & Buerger, 2005), elderly people (Unalan et al., 2015; Wang et al., 2020), patients with multiple sclerosis (Dębska et al., 2020; Glanz et al., 2020), and the general population (Helgeson, 2003).

Despite the importance of the subject, there is no previous research about the psychometric characteristics of the Spanish version of WHOQOL-BREF questionnaire in the Ecuadorian context. Therefore, the objective of this paper was to evaluate the structural validity, convergent validity, internal consistency, and measurement invariance properties of the WHOQOL-BREF in a sample of Ecuadorians.

## Method

### Sample and procedure

An incidental non-probabilistic technique was used to sample the population. The adequate size of the sample for factor analysis will depend on the number of factors, the magnitude of population correlations, and the desired reliability of the correlation coefficients; Tabachnick and Fidell (2012) recommend larger sample sizes. Given that the WHOQOL-BREF is a widely popular tool, we selected 1000 participants from two universities in Cuenca, Ecuador; 13 of them did not complete the questionnaire and were not considered in the analysis. In the University of Cuenca, we recruited students from the Faculty of Medical Sciences which includes the Nursing School and the Medicine School (*n* = 688, 21% were men, average age was 21.26, standard deviation was 2.57) and Faculty of Psychology (*n* = 258, 30% were men, average age was 23.4, standard deviation was 2.2). In the University of Azuay, we recruited students from the Faculty of Economics and Business (*n* = 41, 18% were men, average age and standard deviation were 21.6 and 2.25, respectively). All of them were reached in their classrooms after permission from the authorities was granted. Once an informed consent was obtained, in which the objectives of the investigation were explained, the instruments were handed along with a sociodemographic survey. The application lasted approximately 30 min and was carried out by three psychologists who had practical experience of at least 200 h in psychological evaluation and were trained by the principal investigator for 24 h through instructional classes and role play. The participation of the subjects was anonymous and voluntary without economic or other incentives. Regarding exclusion criteria, we did not consider participants with any severe difficulty in communication (e.g., not being able to read or write) with significant cognitive impairment or suffering from severe visual impairment. Data was collected during December 2017.

The descriptive statistical results of the sample are presented in Table 1. The average age of participants was 21.3 years (SD = 2.5, range = 18–33).

**Table 1:** Sociodemographic characteristics of the sample

Variable		(*n* = 987)	Percentage (%)
Sex	Men	256	25.94%
	Women	731	74.06%
Civil status	Single	877	88.86%
	Married	65	6.59%
	Divorced	2	0.20%
	Common law union	43	4.36%
Ethnic group	Mestizo	955	96.76%
	White	20	2.03%
	Afro-Ecuadorian	4	0.41%
	Indigenous	8	0.81%

### Instruments

#### Questionnaire of sociodemographic characteristics of the participants

An ad hoc questionnaire was developed to characterize the sample based on sociodemographic characteristics: gender, age, marital status, employment status, income, and disability.

#### Spanish version of the Quality of Life Questionnaire (The WHOQOL Group, 1996)

It consists of a total of 26 questions, two general questions about quality of life and satisfaction with the state of health and 24 questions that evaluate four domains: physical health, psychological health, social relations, and environment. The response scales are Likert type with five points. Domain scores can be converted according to a score correction table. Permission for use was requested on the World Health Organization website (*http://www.who.int/substance_abuse/research_tools/whoqolbref/en/*). The Spanish version of the WHOQOL-BREF has been validated in another study (Lucas-Carrasco, 2012). Cultural adaptation could be necessary not only when the questionnaire is used in a different language, but also in this case due to the cultural diversity of Spanish (Lenz et al., 2017; Sousa & Rojjanasrirat, 2011). A panel of experts reviewed the wording of the questions and analyzed whether the questions were understandable to Ecuadorian culture. No changes were suggested. With this version, a pilot test was carried out with 30 adult residents in Cuenca (Ecuador), to ensure understanding of the questionnaire, each participant was asked to report any difficulties they encountered in understanding each of the questions and possible suggestions. No additional modifications were necessary. The sample used in this stage was not included in the data analysis.

#### Rosenberg self-esteem scale (RSE) (Atienza et al., 2000; Rosenberg, 2015)

It consists of 10 items in a 4-point Likert response format (strongly disagree, disagree, agree, strongly agree). Five of the items are presented positively worded and five negatively worded. This instrument has been evaluated and it is appropriate for its use in the Ecuadorian context (Bueno-Pacheco et al., 2020). The internal consistency achieved in this study was a Cronbach’s alpha value of *α* = 0.86.

#### Brief resilience scale (BRS) (Rodríguez-Rey et al., 2016; Smith et al., 2008)

It is a Likert-type scale with six items in a range from 1 (strongly disagree) to 5 (strongly agree). Previous findings support the validity and reliability of this version. This instrument has been evaluated, and it is appropriate for its use in the Ecuadorian context (Peña et al., 2020). The internal consistency obtained in this study showed a Cronbach’s alpha value of *α* = 0.72

#### Functional Social Support questionnaire (Duke-UNC-11) (Bellón Saameño et al., 1996; Broadhead et al., 1988)

It quantitatively evaluates perceived social support and includes two dimensions: confidential social support and affective social support. The 11 items have a Likert response format with scores from 1 to 5 (much less than desired, less than desired, neither much nor less, almost as desired, as much as desired). The range of the total scale goes from 11 to 55 where the higher the score, the greater the perceived social support. This instrument has been evaluated and is appropriate for its use in the Ecuadorian context (Aguilar-Sizer et al., 2021). The internal consistency achieved in this study was a Cronbach’s alpha value of *α* = 0.92

### Data analysis

A descriptive analysis of the WHOQOL-BREF items (average score, standard deviation, skewness, kurtosis, item-total correlation, and Mardia’s multivariate normality test) was used to determine the type of estimator and the type of correlation matrices to be used.

To avoid overfitting problems in factor analysis, the sample was randomly divided into two: the first subsample (*n* = 494) was used to perform exploratory factor analysis (EFA) and exploratory graph analysis (EGA). Confirmatory factor analysis (CFA) was performed in the second subsample (*n* = 493).

In exploratory factor analysis, to determine whether the data matrix can be factored, the Kaiser-Meyer-Olkin coefficient (KMO) was calculated, and the Bartlett sphericity test was performed. If the coefficient is greater than 0.85 and the Bartlett’s null hypothesis is rejected, then factor analysis is carried out. There is no agreement regarding the best way to determine the number of factors to extract; thus, we relied on several criteria that included parallel analysis, very simple structure (VSS), Velicer’s minimum average partial (MAP), and EGA (Golino & Christensen, 2019; Golino & Epskamp, 2017; Horn, 1965; Revelle, 2020). EGA focuses on the estimation of direct relationships between observed variables rather than modeling observed variables as functions of latent common causes. Recent evidence reveals that EGA outperforms other factor extraction methods (H. F. Golino & Epskamp, 2017) and shows promising applications in quality of life research (Kossakowski et al., 2016). Finally, we looked for consistency among the obtained results.

Two options were tested to determine the type of rotation. First, orthogonal rotation was considered as suggested in the original version of WHOQOL-BREF. This option is questioned due to evidence that the orthogonal solution does not fit well in a population whose native language is Spanish (Benitez-Borrego et al., 2014). Second, we used oblimin rotation and checked whether the correlation between factors was at least 0.32 which would reveal an overlap in the variance of the variance between factors of at least 10% (Tabachnick and Fidell (2012). Factor loadings were expected to be at least 0.30, although other scholars consider a value above 0.45 as adequate (Comrey, 2013).

The confirmatory factor analysis (CFA) is performed using diagonally weighted least squares (DWLS) estimator. The goodness of fit of the model was evaluated with different indices such as chi-square, comparative fit index (CFI), Tucker–Lewis index (TLI), standardized root mean squared residual (SRMSR), and root mean square error of approximation (RMSEA). For *CFI* and *TLI*, values between 0.90 and 0.95 were considered to indicate acceptable goodness of fit of a model, while values greater than 0.95 reveal excellent goodness of fit (Hu & Bentler, 1999). For *SRMR* and *RMSEA*, it is considered acceptable when it is below 0.08 and very good when it is less than 0.05 (Steiger & Lind, 1980). Given that we evaluate several models, we also report the Parsimony Normed Fit Index (PNFI) and the Parsimony Global Fit Index (PGFI).

To assess the convergent validity of the questionnaire, the dissatenuated Spearman correlation coefficients were calculated and presented in a correlogram. We hypothesize positive, significant, and moderate correlations (at least 0.4) between the domains of quality of life and self-esteem, confidential social support, affective social support, and the positive dimension of resilience. Likewise, we expect the domains of quality of life to be negatively correlated with the negative dimension of resilience.

The internal consistency of the instrument was evaluated through Cronbach’s alpha (*α*), McDonald’s omega (*ω*) as calculated by Raykov (2001), and hierarchical omega. Scores between 0.7 and 0.8 are considered acceptable, values greater than 0.8 show high consistency, and values greater than 0.9 may indicate redundancy in the questions (Cicchetti, 1994; Tavakol & Dennick, 2011). We also analyze the average extracted variance of each dimension (*AVE*) which value should be 0.5 or higher (Hair et al., 2010).

The invariance of the measurement between men and women was verified using the version of the questionnaire that presents the best validity and internal consistency results. The invariance analysis of measures aims to test the equivalence between the groups. For this, we start testing the equality of population covariance, then we evaluate the configural, metric, residual, scalar, strong means, strict residual, and strict means invariance using Equivalence testing as proposed by Jiang et al. (2017).

All the analysis was conducted in the R software (R Core Team, 2021) using the packages summarytools (Comtois, 2021), polycor (Fox, 2019), GGally (Schloerke et al., 2021), CTT (Willse, 2018), lavaan (Rosseel, 2012), nFactors (Raiche & Magis, 2020), psych (Revelle, 2020). sjPLot (Lüdecke, 2021), REdaS (Maier, 2015), MVN (Korkmaz et al., 2014), EGAnet (Golino & Christensen, 2019), semTools (Jorgensen et al., 2021), semPlot (Epskamp, 2019), and equaltestMI (Jiang et al., 2021)

## Results

The significance of the Mardia's coefficient (*p* < .01) revealed a non-normal multivariate distribution of the data. Items 26, 3, and 4 presented relatively low discrimination coefficients, and items 3 and 4 have the highest kurtosis coefficients among the items with values of 0.98 and 0.8, respectively. See Table 2 for detailed item statistics.

**Table 2:** Descriptive statistics of the items of WHOQOL-BREF

Item	Mean	SD	Skew	Kurtosis	Item discrimination
3. To what extent do you feel that physical pain prevents you from doing what you need to do?	3.32	1.19	− 0.18	− 0.98	0.27
4. How much do you need any medical treatment to function in your daily life?	3.65	1.33	− 0.62	− 0.8	0.22
5. How much do you enjoy life?	3.7	0.85	−0.39	− 0.01	0.63
6. To what extent do you feel your life to be meaningful?	3.86	0.9	− 0.51	− 0.12	0.61
7. How well are you able to concentrate?	3.29	0.76	0.12	0.1	0.48
8. How safe do you feel in your daily life?	3.47	0.8	− 0.07	− 0.11	0.65
9. How healthy is your physical environment?	3.4	0.79	0.01	0.01	0.55
10. Do you have enough energy for everyday life?	3.46	0.86	0.16	− 0.29	0.57
11. Are you able to accept your bodily appearance?	3.76	1.04	− 0.51	− 0.52	0.51
12. Have you enough money to meet your needs?	3	0.91	0.3	0	0.55
13. How available to you is the information that you need in your day-to-day life?	3.53	0.87	0.03	− 0.38	0.59
14. To what extent do you have the opportunity for leisure activities?	2.88	0.91	0.15	− 0.1	0.42
15. How well are you able to get around?	3.73	0.94	− 0.27	− 0.56	0.46
16. How satisfied are you with your sleep?	3.03	0.92	0.26	− 0.06	0.46
17. How satisfied are you with your ability to perform your daily living activities?	3.44	0.84	0.04	0.07	0.61
18. How satisfied are you with your capacity for work?	3.44	0.87	0.02	0	0.59
19. How satisfied are you with yourself?	3.67	0.92	− 0.33	− 0.08	0.66
20. How satisfied are you with your personal relationships?	3.52	0.9	− 0.18	− 0.04	0.61
21. How satisfied are you with your sex life?	3.35	0.98	− 0.04	0.07	0.46
22. How satisfied are you with the support you get from your friends?	3.53	0.92	− 0.31	0.18	0.48
23. How satisfied are you with the conditions of your living place?	3.77	0.95	− 0.31	− 0.44	0.57
24. How satisfied are you with your access to health services?	3.94	0.97	− 0.54	− 0.36	0.5
25. How satisfied are you with your transport?	3.44	1.03	− 0.02	− 0.65	0.47
26. How often do you have negative feelings such as blue mood, despair, anxiety, depression?	3.04	0.93	− 0.11	− 0.43	0.31

### Exploratory factor analysis

Bartlett’s sphericity test (*p* < 0.01) and the KMO criterion (0.91) revealed that the correlation matrix could be factored.

**Fig. 1:**
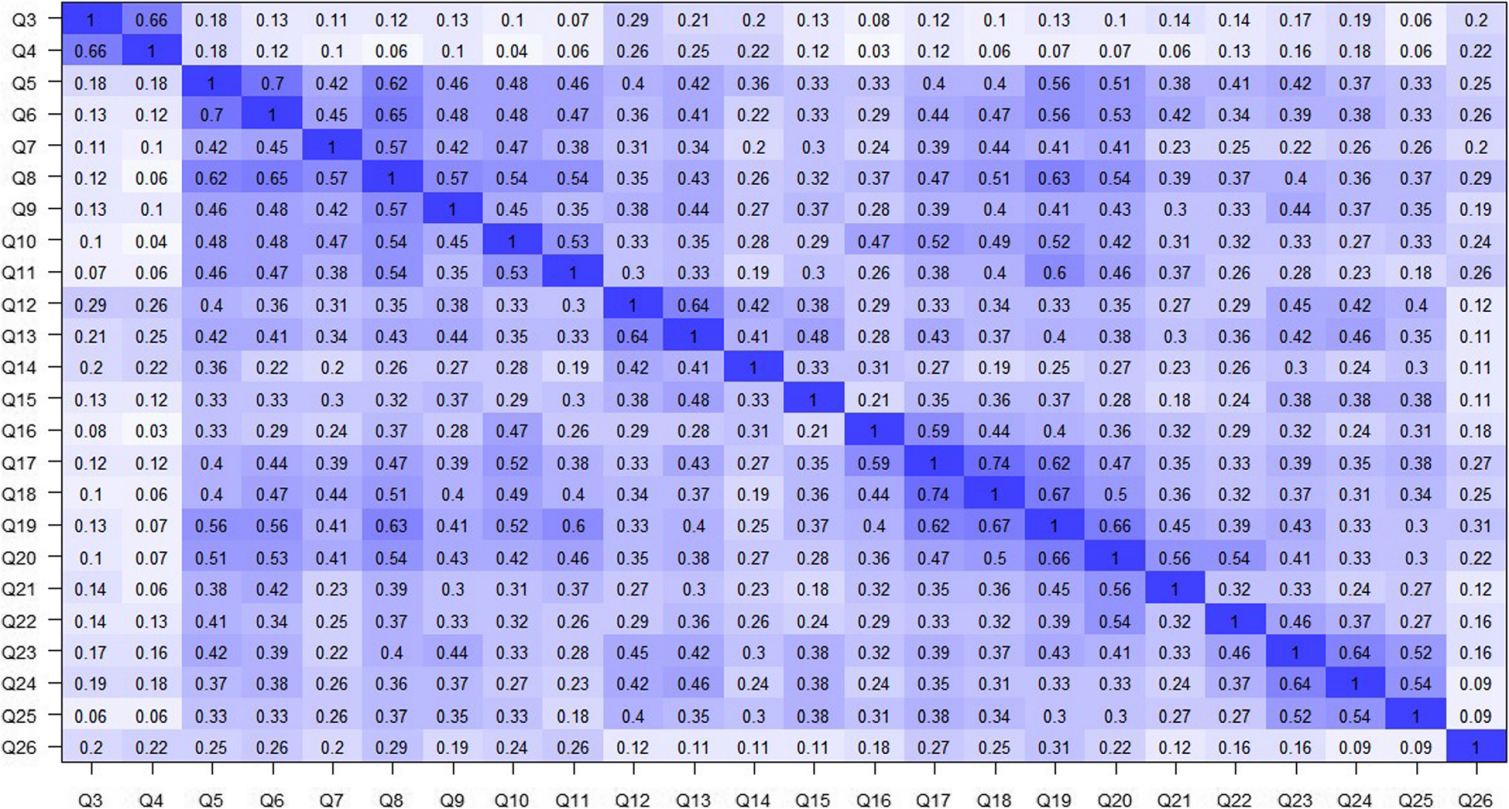
Polychoric correlation matrix of the WHOQOL-BREF

Parallel analysis suggests that the number of factors to be extracted is 8, 3 according to VSS, 2 according to MAP, and 4 according to EGA. Exploratory hierarchical analysis suggests 3 first-order factors and 1 second-order factor. The lack of consistency of these results led us to perform a factor extraction with oblimin rotation and minimum residual estimator extracting 2, 3, 4, and 8 factors. Depending on the number of factors to be extracted, the results show that several items do not discriminate well between the dimensions or have a relatively low factor scoring. For instance, when 8 factors are extracted, we observed that items 15, 21, 22, and 26 score above .30 but below .45. This loading behavior is observed when extracting 4 factors (items 9, 14, 21, 22, 26), 3 factors (items 9, 14, 16, 22), and 2 factors (items 9, 16, 22, 26). The explained variance of these solutions was 62%, 51%, 47%, and 41%, respectively. In the case of the solution of 4 factors, and consistent with other research, we observe that several factor loadings fall under a different factor than those originally suggested by the WHO (see Table 3). A common pattern observed in all the solutions, except the two-factor model, is that items 3 and 4 load into one factor. The problem with a factor that only includes two items is that it merely reflects a correlation between those; however, it is possible to retain such factor if the two items are highly correlated, and the correlation with the other items is relatively low (Worthington & Whittaker, 2006). We explored the polychoric correlation matrix and found that among all the questions, items 3 and 4 have the highest correlation, while they are not strongly correlated with the other questions; therefore, we kept the factor (see Fig. 1).

**Table 3:** Factor loadings for the four-factor solution obtained with EFA

Item	Factor 1	Factor 2	Factor 3	Factor 4
Q3	− 0.01	− 0.01	0.12	0.71
Q4	0	0.01	− 0.05	0.92
Q5	0.7	0.13	− 0.08	0.1
Q6	0.72	0.13	− 0.07	0.01
Q7	0.52	0.02	0.09	0.02
Q8	0.74	0.09	0.01	− 0.02
Q9	0.41	0.25	0.02	0.03
Q10	0.48	− 0.02	0.32	− 0.05
Q11	0.76	− 0.16	0.01	0
Q12	0.03	0.46	0.22	0.17
Q13	0.16	0.52	0.13	0.12
Q14	− 0.04	0.34	0.24	0.13
Q15	0.07	0.46	0.11	0.09
Q16	− 0.09	0.11	0.69	0
Q17	0.06	0.05	0.79	0.05
Q18	0.28	− 0.03	0.58	− 0.06
Q19	0.64	− 0.02	0.23	− 0.01
Q20	0.57	0.16	0.1	− 0.06
Q21	0.38	0.15	0.15	− 0.09
Q22	0.29	0.36	0.01	0.03
Q23	0.14	0.66	− 0.02	0.01
Q24	0.02	0.74	− 0.05	0.02
Q25	− 0.06	0.64	0.12	− 0.09
Q26	0.38	− 0.22	0.08	0.22

**Fig. 2:**
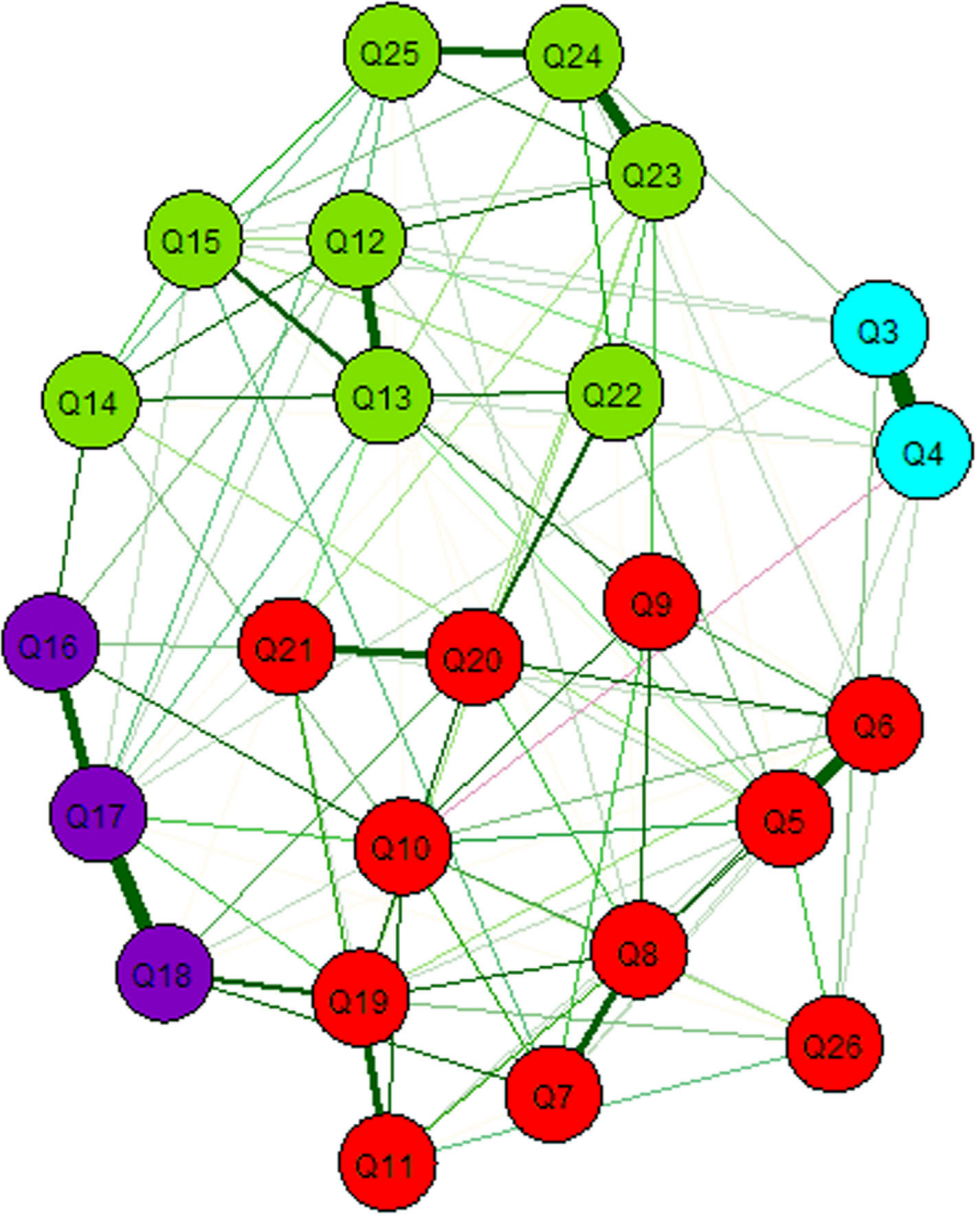
Exploratory graph analysis output

Figure 2 displays the results obtained using exploratory graph analysis.

### Confirmatory factor analysis and internal consistency

Table 4 displays goodness of fit and internal consistency statistics of the original WHOQOL-BREF model (model 1) and its version with correlated dimensions (model 2), a hierarchical version of the original structure (model 3), a 3-factor model (model 4), and the 4-factor model built upon the results of EFA which matched the pattern obtained in the EGA (model 5). The solution of 8 factors is discarded because 2 factors included only two items that did load in more than one factor. Each but model 1 showed adequate values of *CFI* and *TLI*, but only models 4 and 5 presented acceptable values of *RMSEA* and *SRMR*. Additionally, these two models had the lowest chi-squared statistic and the highest PNFI and PGFI.

**Table 4:** Goodness of fit and internal consistency indices of the five models

Model		*Α*	*ω*	*ω*_*H*_	*AVE*	*χ*^*2*^	*df*	*p* value	*CFI*	*TLI*	*SRMR*	*RMSEA*	*RMSEA* 90% CI		*PNFI*	*PGFI*
Model 1	Factor 1	0.724	0.662	0.651	0.651	19332.150	252	< .001	0.352	0.290	0.292	0.392	0.388	0.397	0.319	0.394
	Factor 2	0.805	0.785	0.790	0.790											
	Factor 3	0.723	0.703	0.703	0.703											
	Factor 4	0.848	0.832	0.857	0.857											
Model 2	Factor 1	0.724	0.687	0.717	0.364	1319.750	246	< .001	0.964	0.959	0.075	0.094	0.089	0.099	0.852	0.642
	Factor 2	0.805	0.788	0.800	0.451											
	Factor 3	0.723	0.689	0.697	0.497											
	Factor 4	0.848	0.827	0.843	0.440											
Model 3	Factor 1	0.724	0.687	0.717	0.364	1330.290	248	< .001	0.963	0.959	0.076	0.094	0.089	0.099	0.858	0.647
	Factor 2	0.805	0.788	0.800	0.451											
	Factor 3	0.723	0.689	0.697	0.497											
	Factor 4	0.848	0.827	0.843	0.440											
Model 4	Factor 1	0.829	0.812	0.835	0.435	802.120	249	< .001	0.981	0.979	0.060	0.067	0.062	0.072	0.878	0.658
	Factor 2	0.915	0.901	0.924	0.447											
	Factor 3	0.773	0.726	0.726	0.631											
Model 5	Factor 1	0.893	0.874	0.888	0.460	702.710	246	<. 001	0.984	0.983	0.058	0.061	0.056	0.067	0.870	0.651
	Factor 2	0.773	0.725	0.725	0.632											
	Factor 3	0.831	0.818	0.851	0.411											
	Factor 4	0.804	0.769	0.780	0.612											

*α*, Cronbach’s alpha; *ω*, omega; *ω*_*H*_, hierarchical omega; *AVE*, average variance extracted

Cronbach’s alpha and McDonald’s omega coefficients are fairly similar in model 4 and model 5, but the AVE is slightly higher.

**Fig. 3:**
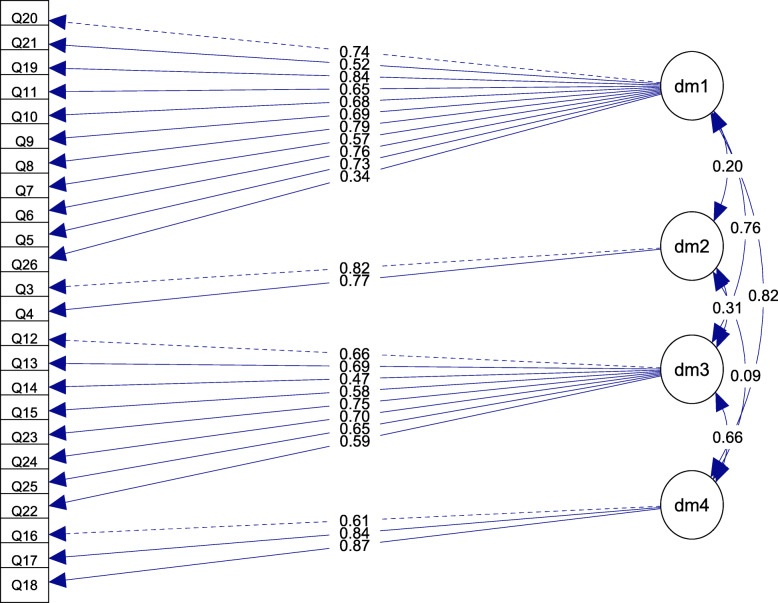
Factor loadings obtained by confirmatory factor analysis

Overall, we conclude that model 5 presents the best results. Figure 3 displays its structure and factor loadings.

### Measurement invariance

See Table 5 for measurement invariance results. The equivalence analysis was performed with several groups using the four correlated first-order model obtained in EGA that had the best structural validity and internal consistency (model 5). Results should be interpreted by comparing the value of *RMSEA*_*t*_ (the T size value of the Wald statistic) with different *RMSEA* adjusted cutoffs. Measurement invariance of the covariance structure is achieved since the values of *RMSEA*_*t*_ of equality of population covariance, configural, metric, residual, and factor variance–covariance models are, most of them, below an adjusted *RMSEA* of 0.08 with the sole exception of the configural model of group 2 whose *RMSEA*_*t*_ is higher than the 0.08 threshold but lower than 0.1. Strong invariance is achieved since the *RMSEA*_*t*_ values are acceptable for the equality of population covariance, configural, metric and scalar models. For strict invariance, it is the *RMSEA*_*t*_ of the strict means model that overpasses the maximum threshold.

**Table 5:** Measurement invariance of the WHOQOL-BREF

Model	*RMSEA*_*t*_	*RMSEA* cutoff values			
		0.01	0.05	0.08	0.1
Equality of population covariance	0.046	0.020	0.056	0.089	0.111
Configural model (group 1: men)	0.057	0.021	0.056	0.088	0.11
Configural model (group 2: women)	0.089	0.021	0.056	0.088	0.11
Metric invariance	0.061	0.04	0.07	0.098	0.117
Residual invariance	0.072	0.038	0.068	0.096	0.116
Factor variance–covariance	0.068	0.05	0.078	0.107	0.126
Scalar invariance	0.075	0.04	0.07	0.098	0.117
Strong means invariance	0.146	0.069	0.095	0.124	0.143
Strict residual invariance	0.073	0.038	0.068	0.096	0.116
Strict means invariance	0.146	0.069	0.095	0.124	0.143

### Convergent validity.

**Fig. 4:**
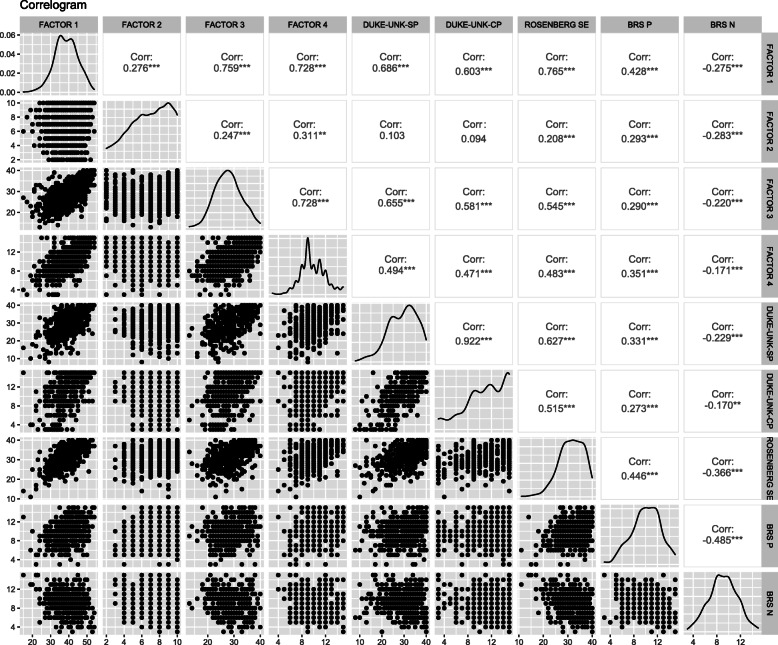
Correlogram to assess convergent validity

A total of three instruments and five dimensions were used to determine the convergent validity of the four dimensions of the WHOQOL-BREF (see Fig. 4). All correlations were statistically significant (*p* < .001) except one factor that did not correlate with social support.

## Discussion

The psychometric analysis does not allow us to support the original uncorrelated structure of the WHOQOL-BREF. Despite a great improvement and good psychometric properties are observed when factors are allowed to correlate, an alternative four-factor structure is proposed for it presents better psychometric properties.

The results of other investigations revealed that the instrument could have a factor structure different from the one originally proposed. Variations have also been identified in relation to the original four-domain model in Spanish-speaking populations from the following countries: Costa Rica, Peru, Mexico, Cuba, Paraguay, Argentina, Colombia, Spain, and Chile (Benitez-Borrego et al., 2014; Benítez-Borrego et al., 2016; Huerta et al., 2017; Urzúa, & A.,, & Caqueo-Urízar, A., 2013). In other non-Spanish–speaking countries, for example, in Brazil, the original structure of the WHOQOL-BREF could not be replicated. The discrepancies observed could be due to the following characteristics of the population involved in the study: active working age and being relatively healthy (Moreno et al., 2006). Another study in Portugal involved 403 participants recruited from mental health centers could not confirm the structure of four factors of the instrument (Oliveira et al., 2016). In Nigeria, a study involving 118 recently recovered psychotic patients found that eight domains have better structural integrity indices in the confirmatory factor analysis than the WHO four-domain model (Ohaeri et al., 2004). Besides, the absence of psychometric equivalence between the Iranian and Belgian population would signal that there could be different ways of perceiving the quality of life (Theuns et al., 2010). Another study with culturally heterogeneous samples compared the English, Malay, and Chinese versions of the WHOQOL-BREF, finding partial psychometric equivalence. Although the English and Malay versions were equivalent in all domains, the English and Chinese versions were equal only in the physical and environmental domains but not for the psychological and social domains (Cheung et al., 2019).

These findings could be explained, in the first place, by the fact that the original structure of the WHOQOL-BREF fits better in samples that contain a wide range of age and health status, since this questionnaire was initially developed and validated using large clinical and nonclinical populations (Suárez et al., 2018). Second, these findings could be explained by the nationality of the participants since, despite using a common language, there are differences in cultural, historical, and social variables between Spanish-speaking countries that could be influencing the individual’s perception of the different domains of the quality of life (Benítez-Borrego et al., 2016). Third, young people’s conceptions about quality of life give importance to positive expectations for the future, social relationships, and finding an interesting job, whereas adults prioritize other aspects (Saxena et al., 2001).

Considering these results and the approaches of Nisbett (2004) who recognizes that members of different cultures differ in their fundamental beliefs about the nature of the world, the western way of seeing the world can be related to ancient Greek philosophy whose concepts are strange in other cultures. Conceptually, it is conceivable to consider the people of a culture to have a holistic sense of quality of life, while in other cultures, there are more differentiated subjective evaluations of the quality of life and well-being in terms of domains. Although Nisbett’s work focuses on the comparison between western (mainly American) and Asian populations, it is reasonable to think that instruments designed in the West need a careful review of content before being applied to other nations, including Latin American countries. This is reflected both in the exploratory factor analysis and in the confirmatory factor analysis.

In exploratory factor analysis, the techniques used to extract factors offered different results. Furthermore, like other research, the analysis reveals that the dimensions are correlated, which suggests a high degree of overlap between domains or even the presence of a general dimension of quality of life. In this paper, we found out that the dimensions are correlated as well, and in addition, we explored a possible hierarchical model with a second-order factor that represent a general dimension. The finding of a one-dimensional solution of WHOQOL-BREF is found in the literature, although it is based on data from a shorter version of eight items (Snell et al., 2016).

In confirmatory factor analysis, we evaluated the structural validity of five different models of the WHOQOL-BREF. Only the version of orthogonal factors showed undisputable poor goodness of fit indices that improved after allowing factors to correlate (model 2). In models 2 and 3, the values of *CFI*, *TLI*, and *SRMR* revealed good fit, but the *RMSEA* did not (*RMSEA* > 0.08). The two models derived from exploratory factor analysis (models 4 and 5) had good fit, with the latter having slightly better outputs. More importantly, two different strategies, EFA and EGA, lead to the same factor structure presented in model 5; this model, displayed in Fig. 3, presented the best psychometric properties and includes a 2-item factor (items 3 and 4). Other projects undertaken in Thailand, New Zealand, and China informed that the items 3 and 4 were not considered as relevant or appropriate for samples of young university students with a low proportion of self-reported health problems (Krägeloh et al., 2011; Li et al., 2009; Zhang et al., 2012). Consequently, we adhere to the recommendation that these items should be carefully interpreted with samples of young individuals that are less likely to have physical health problems.

The model we propose includes four dimensions that account for psychological (items 5, 6, 7, 8, 9,10, 11, 19, 20, 21, 26), a dimension with two items (items 3 and 4), environmental (items 12, 13, 14, 15, 22, 23, 24, 25), and physical health (items 16, 17, 18). In the case of the first domain, the clustering of items, may be the result of the influence of certain sociocultural variables not analyzed in this study, such as the importance that the participants in this region may attribute to relationships and ties for psychological health.

A research study involving 4804 respondents from 15 places, from 14 developed and developing countries and 12 languages found that younger adults rated more items as significantly more important to them than the older adults. These items included psychological aspects (e.g., to think clearly, to feel hopeful) and social/work aspects (e.g., relationship with other people, to be able to work) (Saxena et al., 2001). Therefore, for a comparative and international perspective research, the information on the gender- and age-specific items for WHOQOL-BREF is likely to be useful not only in clarifying the concept of quality of life but also in paying attention to these aspects in interpreting the results obtained using generic measures of quality of life (Saxena et al., 2001).

Regarding measurement invariance, we evaluated different models by adding constraints. First, we perform a hypothesis test to determine whether the population variance matrices are equal. A result of *RMSEA*_*t*_ lower than the lowest cutoff would support the overall invariance of the instrument. In this case, the obtained value is higher than the 0.01 but lower than the 0.05 cutoff which leads us to further add constraints to evaluate the invariance of the instrument. Second, we examine the configural invariance and find that the goodness of fit is fair in group 1 but mediocre in group 2. The models with additional constraints show fair goodness of fit, except for strong means invariance and strict means invariance unless we are willing to tolerate a large *RMSEA*. We can conclude that strong invariance (configural, metric, and scalar) and strict (configural, metric, scalar, and residual) invariance are achieved. The high values of *RMSEA*_*t*_ for strict and strong means invariance demand a more detailed examination on the source of the lack of invariance.

The convergent validity analysis reveals moderate and significant correlations between the four dimensions proposed related to quality of life: self-esteem (van Leeuwen et al., 2012), resilience (Aranguren, 2017), and social support (Yamout et al., 2013). The magnitude of the correlation is lower than expected for the second factor that groups items 3 and 4. However, as noted before, we recommend caution with the interpretation of this factor.

All the evaluated models present good internal consistency in each of its dimensions. Nevertheless, model 5 has the highest coefficients and highest values of *AVE*.

For practical purposes, the original version of the WHOQOL-BREF can be used until more evidence of the alternative model is provided. When young healthy adults are being screened, items 3 and 4 should be interpreted with caution.

Some limitations of this research deserve attention as they guide the proper interpretation of the results. First, the cross-sectional design of this research should be considered, so the predictive or test–retest validity of this questionnaire is not analyzed. Second, the sample was drawn from two universities; the population surveyed is not demographically diverse enough to be representative of all Ecuadorian population, rather, representative of young adults.

## Conclusion

We find that the original uncorrelated factor structure of the WHOQOL-BREF does not present adequate psychometric properties; however, when factors are allowed to correlate, we observe good structural validity and internal consistency. Given the lack of consensus on the cross-cultural replicability of the original internal structure in other types of samples of young people or general population with different socioeconomic and health conditions, we propose a factor structure obtained by two strategies, EFA and EGA, that shows good structural validity, internal consistency, and invariance. The factor structure we propose contributes to improve the results of professional assessment and to the scarce assessment literature in the Ecuadorian context. For practical purposes, researchers may use the original four-factor with correlated factors because the model proposed in this paper needs to be examined in samples with other sociodemographic characteristics to further assure its external validity.

## Data Availability

Available upon request

## Abbreviations

QOL: Quality of life
WHO: World Health Organization
WHOQOL-BREF: The World Health Organization Quality of Life questionnaire, short version
RSE: Rosenberg self-esteem scale
BRS: Brief resilience scale
Duke-UNC-11: Functional Social Support questionnaire
KMO: Kaiser-Meyer-Olkin
VSS: Very simple structure
MAP: Veliger’s minimum average partial
EGA: Exploratory graph analysis
DWLS: Diagonally weighted least squares
CFI: Comparative fit index
TLI: Tucker-Lewis index
SRMSR: Standardized root mean squared residual
RMSEA: Root mean square error of approximation
EFA: Exploratory factor analysis
CFA: Confirmatory factor analysis
AVE: Average extracted variance

## References

[CR1] Aguilar-Sizer M, Lima-Castro S, Arias-Medina P, Peña-Contreras E, Bueno-Pacheco A, Marcela C-V (2021). Propiedades Psicométricas del Cuestionario de Apoyo Social Funcional Duke-UNK-11 en una Muestra de Adultos Ecuatorianos. Revista Eureka.

[CR2] Aranguren P (2017). Resilience, pain and quality of life in people with physical disabilities: A systematic review. European Psychiatry.

[CR3] Atienza F, Moreno Y, Balaguer I (2000). Análisis de la dimensionalidad de la Escala de Autoestima de Rosenberg en una muestra de adolescentes valencianos. *Revista de Psicología*. Universitas Tarraconensis.

[CR4] Barneveld PS, Swaab H, Fagel S, Van Engeland H, De Sonneville LMJ (2014). Quality of life: A case-controlled long-term follow-up study, comparing young high-functioning adults with autism spectrum disorders with adults with other psychiatric disorders diagnosed in childhood. Comprehensive Psychiatry.

[CR5] Bellón Saameño, J. A., Delgado Sánchez, A., Luna del Castillo, J. D., & Lardelli Claret, P. (1996). Validity and reliability of the Duke-UNC-11 questionnaire of functional social support. *Atencion Primaria /Sociedad Española de Medicina de Familia y Comunitaria*, *18*(4), 153–163. Retrieved from f:%5CArt?culos en PDF%5C10549 AtenPrimaria Bellon Saame?o.pdf 8962994

[CR6] Benitez-Borrego S, Guàrdia-Olmos J, Urzúa-Morales A (2014). Factorial structural analysis of the Spanish version of WHOQOL-BREF: an exploratory structural equation model study. Quality of Life Research: An International Journal of Quality of Life Aspects of Treatment, Care and Rehabilitation.

[CR7] Benítez-Borrego S, Mancho-Fora N, Farràs-Permanyer L, Urzúa-Morales A, Guàrdia-Olmos J (2016). Differential item functioning of WHOQOL-BREF in nine Iberoamerican countries. Revista Iberoamericana de Psicologia y Salud.

[CR8] Bowden A, Fox-Rushby JA (2003). A systematic and critical review of the process of translation and adaptation of generic health-related quality of life measures in Africa, Asia, Eastern Europe, the Middle East, South America. Social Science and Medicine.

[CR9] Broadhead WE, Gehlbach SH, de Gruy FV, Kaplan BH (1988). The Duke-UNC functional social support questionnaire: Measurement of social support in family medicine patients. Medical Care.

[CR10] Bueno-Pacheco A, Lima-Castro S, Arias-Medina P, Peña-Contreras E, Aguilar-Sizer M, Cabrera-Vélez M (2020). Estructura Factorial, Invarianza y Propiedades Psicométricas de la Escala de Autoestima de Rosenberg en el Contexto Ecuatoriano. Revista Iberoamericana de Diagnóstico y Evaluación – e Avaliação Psicológica.

[CR11] Burgess AF, Gutstein SE (2007). Quality of life for people with autism: Raising the standard for evaluating successful outcomes. Child and Adolescent Mental Health.

[CR12] Cheung YB, Yeo KK, Chong KJ, Khoo EYH, Wee HL (2019). Measurement equivalence of the English, Chinese and Malay versions of the World Health Organization Quality of Life (WHOQOL-BREF) questionnaires. Health and Quality of Life Outcomes.

[CR13] Cicchetti DV (1994). Guidelines, criteria, and rules of thumb for evaluating normed and standardized assessment instruments in psychology. Psychological Assessment.

[CR14] Comrey, A. L. (2013). *A first course in factor analysis*. *A first course in factor aalysis. Psychology press.*10.4324/9781315827506.

[CR15] Comtois, D. (2021). summarytools: Tools to quickly and neatly summarize data (R package version 0.9.9) [Computer software]. The Comprehensive R Archive Network. Available from https://CRAN.R-project.org/package=summarytools

[CR16] Crocker TF, Smith JK, Skevington SM (2015). Family and professionals underestimate quality of life across diverse cultures and health conditions: Systematic review. Journal of Clinical Epidemiology.

[CR17] Cummins, R. A., & Lau, A. L. (2006). Using health and subjective wellbeing for quality of life measurement: A review. In L. Bauld (Ed.), *Social Policy Review 18: Analysis and Debate in Social Policy, 2006*. Bristol: The Policy Press.

[CR18] Dębska G, Milaniak I, Skorupska-Król A (2020). The quality of life as a predictor of social support for multiple sclerosis patients and caregivers. The Journal of Neuroscience Nursing.

[CR19] Epskamp, S. (2019). semPlot: Path diagrams and visual analysis of various sem packages' Output (R package version 1.1.2) [Computer software]. The Comprehensive R Archive Network. Available from https://CRAN.R-project.org/package=semPlot

[CR20] Fox, J. (2019). polycor: Polychoric and polyserial correlations (R package version 0.7-10) [Computer software]. The Comprehensive R Archive Network. Available from https://CRAN.R-project.org/package=polycor

[CR21] Glanz BI, Zurawski J, Gonzalez CT, Shamah R, Ratajska A, Chitnis T, Weiner HL, Healy BC (2020). Comparison of health-related quality of life across treatment groups in individuals with multiple sclerosis. Multiple Sclerosis and Related Disorders.

[CR22] Golino, H., & Christensen, A. P. (2019). EGAnet: Exploratory graph analysis – a framework for estimating the number of dimensions in multivariate data using network psychometrics (R package version 0.9.8) [Computer software]. The Comprehensive R Archive Network. Available from https://CRAN.R-Project.Org/Package=EGAnet.

[CR23] Golino, H. F., & Epskamp, S. (2017). Exploratory graph analysis: A new approach for estimating the number of dimensions in psychological research. *PLoS ONE*, *12*(6), e0174035. 10.1371/journal.pone.0174035. 10.1371/journal.pone.0174035 PMC5465941 28594839

[CR24] Hair, J. F., Black, W. C., Babin, B. J. & Anderson, R. E. (2010). *Multivariate data analysis* (7th edition). Upper Saddle River: Pearson Education.

[CR25] Hambleton, R. K., Merenda, P. F., & Spielberger, C. D. (2004). *Adapting educational and psychological tests for cross-cultural assessment*. *Adapting Educational and Psychological Tests for Cross-Cultural Assessment.*10.4324/9781410611758.

[CR26] Harper A, Power M, Orley J, Herrman H, Schofield H, Murphy B (1998). Development of the World Health Organization WHOQOL-BREF Quality of Life Assessment. Psychological Medicine.

[CR27] Helgeson VS (2003). Social support and quality of life. Quality of Life Research.

[CR28] Hemati Z, Kiani D (2016). The relationship between self-esteem and quality of life of patients with idiopathic thrombocytopenic purpura at Isfahan’s Sayed Al-Shohada Hospital, Iran, in 2013. International Journal of Hematology-Oncology and Stem Cell Research.

[CR29] Horn JL (1965). A rationale and test for the number of factors in factor analysis. Psychometrika.

[CR30] Hu LT, Bentler PM (1999). Cutoff criteria for fit indexes in covariance structure analysis: Conventional criteria versus new alternatives. Structural Equation Modeling.

[CR31] Huerta JAL, Romo RAG, Tayabas JMT (2017). Propiedades Psicométricas de la Versión en Español de la Escala de Calidad de Vida WHOQOL BREF en una Muestra de Adultos Mexicanos. Revista Iberoamericana de Diagnostico y Evaluacion Psicologica.

[CR32] Jang Y, Hsieh CL, Wang YH, Wu YH (2004). A validity study of the WHOQOL-BREF assessment in persons with traumatic spinal cord injury. Archives of Physical Medicine and Rehabilitation.

[CR33] Jiang, G., Mai, Y., & Yuan, K. H. (2017). Advances in measurement invariance and mean comparison of latent variables: Equivalence testing and a projection-based approach. *Frontiers in Psychology*, *8*(OCT). 10.3389/fpsyg.2017.01823. 10.3389/fpsyg.2017.01823 PMC5660858 29114237

[CR34] Jiang, G., Mai, Y., & Yuan, K. H. (2021). equaltestMI: Examine measurement invariance via equivalence testing and Projection Method (R package version 0.6.1) [Computer software]. The Comprehensive R Archive Network. Available from https://CRAN.R-project.org/package=equaltestMI

[CR35] Jorgensen, T., Pornprasertmanit, S., Schoemann, A., & Rosseel, Y. (2021). semTools: Useful tools for structural equation modeling (R package version 0.5-4) [Computer software]. The Comprehensive R Archive Network. Available from https://CRAN.R-project.org/package=semTools

[CR36] Korkmaz, S., Goksuluk, D., & Zararsiz, G. (2014). MVN: An r package for assessing multivariate normality [Computer software]. The Comprehensive R Archive Network. Available from https://journal.r-project.org/archive/2014-2/korkmaz-goksuluk-zararsiz.pdf

[CR37] Kossakowski JJ, Epskamp S, Kieffer JM, van Borkulo CD, Rhemtulla M, Borsboom D (2016). The application of a network approach to Health-Related Quality of Life (HRQoL): Introducing a new method for assessing HRQoL in healthy adults and cancer patients. Quality of Life Research.

[CR38] Krägeloh, C. U., Henning, M. A., Hawken, S. J., Zhao, Y., Shepherd, D., & Billington, R. (2011). Validation of the WHOQOL-BREF quality of life questionnaire for use with medical students. *Education for Health: Change in Learning and Practice*, *24*(2). 22081657

[CR39] Kuehner, C., & Buerger, C. (2005). Determinants of subjective quality of life in depressed patients: The role of self-esteem, response styles, and social support. *Journal of Affective Disorders*, *86*(2–3), 205–213. 10.1016/j.jad.2005.01.014, 213 10.1016/j.jad.2005.01.014 15935240

[CR40] Lenz AS, Soler IG, Dell’Aquilla J, Uribe PM (2017). Translation and cross-cultural adaptation of assessments for use in counseling research. Measurement and Evaluation in Counseling and Development.

[CR41] Li K, Kay NS, Nokkaew N (2009). The performance of the World Health Organization’s WHOQOL-BREF in assessing the quality of life of Thai college students. Social Indicators Research.

[CR42] Lucas-Carrasco R (2012). The WHO quality of life (WHOQOL) questionnaire: Spanish development and validation studies. Quality of Life Research.

[CR43] Lüdecke, D. (2021). sjPlot: Data visualization for statistics in social science (R package version 2.8.7) [Computer software]. The Comprehensive R Archive Network. Available from https://CRAN.R-project.org/package=sjPlot

[CR44] Maier, M. (2015). Companion Package to the Book “R: Einführung durchangewandte Statistik” (R package version 0.9.3) [Computer software]. The Comprehensive R Archive Network. Available from http://CRAN.R-project.org/package=REdaS.

[CR45] Min SK, Kim KI, Lee CI, Jung YC, Suh SY, Kim DK (2002). Development of the Korean versions of WHO Quality of Life scale and WHOQOL-BREF. Quality of Life Research.

[CR46] Moreno AB, Faerstein E, Werneck GL, Lopes CS, Chor D (2006). Psychometric properties of the World Health Organization abbreviated instrument for quality of life assessment in the Pró-Saúde Study. Cadernos de Saude Publica.

[CR47] Nisbett, R. (2004). *The geography of thought: How Asians and Westerners think differently... and why*. New York: The Free Press.

[CR48] Ohaeri JU, Awadalla AW, El-Abassi AHM, Jacob A (2007). Confirmatory factor analytical study of the WHOQOL-Bref: Experience with Sudanese general population and psychiatric samples. BMC Medical Research Methodology.

[CR49] Ohaeri JU, Olusina AK, Al-Abassi AHM (2004). Factor analytical study of the short version of the World Health Organization quality of life instrument. Psychopathology.

[CR50] Oliveira SEH, Carvalho H, Esteves F (2016). Toward an understanding of the quality of life construct: Validity and reliability of the WHOQOL-Bref in a psychiatric sample. Psychiatry Research.

[CR51] Peña EK, Lima S, Arias P, Bueno A, Aguilar M, Cabrera M (2020). Propiedades psicométricas de la Escala Breve de Resiliencia (BRS) en el contexto ecuatoriano. Revista Evaluar.

[CR52] Perera HN, Izadikhah Z, O’Connor P, McIlveen P (2018). Resolving dimensionality problems with WHOQOL-BREF item responses. Assessment.

[CR53] R Core Team (2021). R: A language and environment for statistical computing (version 4.1.1) [Computer software]. Vienna, Austria. Available from https://www.R-project.org/.

[CR54] Raiche, G., & Magis, D. (2020). nFactors: Parallel analysis and other nongraphical solutions to the Cattell Scree Test (R package version 2.4.1) [Computer software]. The Comprehensive R Archive Network. Available from https://CRAN.R-project.org/package=nFactors.

[CR55] Raykov T (2001). Estimation of congeneric scale reliability using covariance structure analysis with nonlinear constraints. British Journal of Mathematical and Statistical Psychology.

[CR56] Revelle, M. W. (2020). *psych: Procedures for personality and psychological research (R package)*.

[CR57] Ristevska-Dimitrоvska G, Filov I, Rajchanovska D, Stefanovski P, Dejanova B (2015). Resilience and quality of life in breast cancer patients. Open Access Macedonian Journal of Medical Sciences.

[CR58] Rodríguez-Rey R, Alonso-Tapia J, Hernansaiz-Garrido H (2016). Research on translations of tests: Reliability and validity of the brief resilience scale (BRS) Spanish version. Psychological Assessment.

[CR59] Rosenberg, M. (2015). *Society and the adolescent self-image*. *Society and the Adolescent Self-Image*. Princeton University press. 10.1177/003803856900300250.

[CR60] Rosseel, Y. (2012). lavaan: An R package for structural equation modeling (R package version 0.6-8) [Computer software]. The Comprehensive R Archive Network. Available from https://www.jstatsoft.org/v48/i02/.

[CR61] Saxena S, Carlson D, Billington R, Orley J (2001). The WHO quality of life assessment instrument (WHOQOL-Bref): The importance of its items for cross-cultural research. Quality of Life Research.

[CR62] Schloerke, B., Cook, D., Larmarange, J., Briatte, F., Marbach, M., Thoen, E., Elberg, A., & Crowley, J. (2021). GGally: Extension to 'ggplot2' (R package version 2.1.1) [Computer software]. The Comprehensive R Archive Network. Available from https://CRAN.R-project.org/package=GGally.

[CR63] Skevington SM, Epton T (2018). How will the sustainable development goals deliver changes in well-being? A systematic review and meta-analysis to investigate whether WHOQOL-BREF scores respond to change. BMJ Global Health.

[CR64] Skevington SM, Lotfy M, O’Connell KA (2004). The World Health Organization’s WHOQOL-BREF quality of life assessment: Psychometric properties and results of the international field trial. A report from the WHOQOL Group. Quality of Life Research.

[CR65] Smith BW, Dalen J, Wiggins K, Tooley E, Christopher P, Bernard J (2008). The brief resilience scale: Assessing the ability to bounce back. International Journal of Behavioral Medicine.

[CR66] Snell DL, Siegert RJ, Surgenor LJ, Dunn JA, Hooper GJ (2016). Evaluating quality of life outcomes following joint replacement: Psychometric evaluation of a short form of the WHOQOL-BREF. Quality of Life Research.

[CR67] Sosnowski R, Kulpa M, Ziȩtalewicz U, Wolski JK, Nowakowski R, Bakuła R, Demkow T (2017). Basic issues concerning health-related quality of life. Central European Journal of Urology.

[CR68] Sousa VD, Rojjanasrirat W (2011). Translation, adaptation and validation of instruments or scales for use in cross-cultural health care research: A clear and user-friendly guideline. Journal of Evaluation in Clinical Practice.

[CR69] Steiger, J. H., & Lind, J. C. (1980). Statistically based tests for the numbers of factors. In *Annual meeting of the Psychometric Society*.

[CR70] Suárez L, Tay B, Abdullah F (2018). Psychometric properties of the World Health Organization WHOQOL-BREF Quality of Life assessment in Singapore. Quality of Life Research.

[CR71] Tabachnick, B. G., & Fidell, L. S. (2012). Using multivariate statistics (6th ed.). New York: Harper and Row.

[CR72] Taillefer MC, Dupuis G, Roberge MA, LeMay S (2003). Health-related quality of life models: Systematic review of the literature. Social Indicators Research.

[CR73] Tavakol M, Dennick R (2011). Making sense of Cronbach’s alpha. International Journal of Medical Education.

[CR74] Tavares DM, Matias TG, Ferreira PC, Pegorari MS, Nascimento JS, Paiva MM (2016). Quality of life and self-esteem among the elderly in the community. Ciencia e Saude Coletiva.

[CR75] The WHOQOL Group. (1996). WHOQOL-BREF: Introduction, administration, scoring and generic version of the assessment. Geneva: World Health Organization.

[CR76] Theuns P, Hofmans J, Mazaheri M, Van Acker F, Bernheim JL (2010). Cross-national comparability of the WHOQOL-BREF: A measurement invariance approach. Quality of Life Research.

[CR77] Unalan D, Gocer S, Basturk M, Baydur H, Ozturk A (2015). Coincidence of low social support and high depressive score on quality of life in elderly. European Geriatric Medicine.

[CR78] Urzúa M, A., & Caqueo-Urízar, A. (2013). Estructura factorial y valores de referencia del WHOQOL-BREF en población adulta chilena. Revista Medica de Chile.

[CR79] van Leeuwen, C. M. C., Kraaijeveld, S., Lindeman, E., & Post, M. W. M. (2012). Associations between psychological factors and quality of life ratings in persons with spinal cord injury: A systematic review. *Spinal Cord.*10.1038/sc.2011.120. 10.1038/sc.2011.120 22042298

[CR80] Wang J, Xue J, Jiang Y, Zhu T, Chen S (2020). Mediating effects of depressive symptoms on social support and quality of life among rural older Chinese. Health and Quality of Life Outcomes.

[CR81] Willse, J. (2018). CTT: Classical test theory functions (R package version 2.3.3) [Computer software]. The Comprehensive R Archive Network. Available from https://CRAN.R-project.org/package=CTT.

[CR82] Worthington RL, Whittaker TA (2006). Scale development research: A content analysis and recommendations for best practices. The Counseling Psychologist.

[CR83] Yamout B, Issa Z, Herlopian A, El Bejjani M, Khalifa A, Ghadieh AS, Habib RH (2013). Predictors of quality of life among multiple sclerosis patients: A comprehensive analysis. European Journal of Neurology.

[CR84] Yao G, Chung CW, Yu CF, Der Wang J (2002). Development and verification of validity and reliability of the WHOQOL-BREF Taiwan version. Journal of the Formosan Medical Association.

[CR85] Yazdi-Ravandi S, Taslimi Z, Saberi H, Shams J, Osanlo S, Nori G, Haghparast A (2013). The role of resilience and age on quality of life in patients with pain disorders. Basic and Clinical Neuroscience.

[CR86] Zhang, Y., Qu, B., Lun, S., Wang, D., Guo, Y., & Liu, J. (2012). Quality of Life of medical students in China: A study using the WHOQOL-BREF. *PLoS ONE*, *7*(11). 10.1371/journal.pone.0049714. 10.1371/journal.pone.0049714 PMC3507917 23209595

